# Evaluation of external stabilization of type II odontoid fractures in geriatric patients—An experimental study on a newly developed cadaveric trauma model

**DOI:** 10.1371/journal.pone.0260414

**Published:** 2021-11-29

**Authors:** Matthias K. Jung, Gregor V. R. von Ehrlich-Treuenstätt, Andreas L. Jung, Holger Keil, Paul A. Grützner, Niko R. E. Schneider, Michael Kreinest

**Affiliations:** 1 BG Trauma Center Ludwigshafen, Clinic for Trauma and Orthopaedic Surgery, University of Heidelberg, Ludwigshafen on the Rhine, Germany; 2 Clinic for Trauma and Orthopaedic Surgery, Universitätsklinikum Erlangen, Erlangen, Germany; 3 Clinic of Anesthesiology, University of Heidelberg, Heidelberg, Germany; University Hospital Zurich, SWITZERLAND

## Abstract

**Background:**

Along with the growing geriatric population, the number of odontoid fractures is steadily increasing. However, the effectiveness of immobilizing geriatric odontoid fractures using a cervical collar has been questioned. The aim of the present study is to analyze the physiological and pathological motion in odontoid fractures and to assess limitation of motion in the cervical spine when applying a cervical collar.

**Methods:**

Motion analysis was performed with wireless motion tracker on unfixed geriatric human cadavers. First, a new geriatric type II odontoid fracture model was developed. In this model, the type II odontoid fracture is operated via a transoral approach. The physiological and pathological flexion and lateral bending of the cervical spine resulting from this procedure was measured. The resulting motion after external stabilization using a cervical collar was analyzed.

**Results:**

The new geriatric type II odontoid fracture model was successfully established using seven unfixed human cadavers. The pathological flexion of the cervical spine was significantly increased compared to the physiological flexion (*p* = 0.027). Furthermore, the flexion was significantly reduced when a cervical collar was applied. In case of flexion the mean remaining motion was significantly reduced (*p* = 0.0017) from 41° to 14°. For lateral bending the mean remaining motion was significantly reduced (*p* = 0.0137) from 48° to 18°.

**Conclusions:**

In case of type II odontoid fracture, flexion and lateral bending of the cervical spine are increased due to spinal instability. Thus, if an odontoid fracture is suspected in geriatric patients, the application of a cervical collar should always be considered since external stabilization can significantly reduce flexion and lateral bending.

## Introduction

In the geriatric population, the most common fractures of the cervical spine are odontoid fractures, accounting for more than 50% of total cervical spine fractures [1, 2]. In 1974, Anderson and D’Alonzo developed a system for classification of odontoid fracture that is still in use today [3]. This classification distinguishes three types of odontoid fracture: a type I odontoid fracture is located at the tip of the dens axis; in type II, the fracture line runs through the base of the dens axis; and in type III, the corpus of the dens axis is affected. Type II odontoid fractures are the most common, representing up to 68% of all odontoid fractures [4, 5]. Poor bone quality together with pre-existing conditions, such as osteopenia and osteoporosis, are considered to be the main reasons accounting for odontoid fractures in the geriatric population [6]. Thus, unlike odontoid fractures in young patients, these injuries in the geriatric population are mainly caused by minor trauma and falls at home [7–9]. These low-impact trauma mechanisms are one factor that may explain why these patients are often under-triaged by Emergency Medical Service personnel (EMS) at the scene of an accident as well as in the admitted hospital [10, 11]. Furthermore, in contrast to young patients, a typical geriatric facial structure is described [12, 13]. It is unclear whether this change in facial structure has an influence on the remaining motion after external immobilization of the cervical spine. Due to demographic changes, the number of geriatric trauma patients is constantly increasing [14]. Geriatric trauma patients have a higher mortality rate [15] and, therefore, require special attention.

The external stabilization of the cervical spine using a cervical collar as part of pre-hospital trauma care remains controversial [16–20]. According to literature, the geriatric population is particularly exposed to an increased risk of complications if a cervical collar is used [21, 22]. Minor negative effects such as pain, discomfort [23, 24], or pressure ulceration [25, 26] have been described, but major negative effects such as restrictions in respiratory function [27], difficult airway management [28], increased intracranial pressure [29, 30], and even the compression of the dural sac [31] have also been described. These negative effects have led to a general doubt about the effectiveness of a cervical collar in the case of an injury to the upper cervical spine [31–35]. At least, these doubts have resulted in the development of current guidelines that recommend the omission of cervical collars in trauma patients [32, 34, 36]. However, these recommendations are in conflict with a number of international guidelines and immobilization protocols that still recommend external stabilization of the cervical spine of trauma patients using a cervical collar [18, 37–39], since the benefit of a cervical collar is thought to outweigh its negative effects.

The aim of the present study was to analyze whether the application of a cervical collar can reduce cervical spine motion in geriatric patients with a type II odontoid fracture. For this purpose, a new cadaveric trauma model was established, and the extent of physiological cervical spine flexion and lateral bending as well as the extent of pathological cervical spine motion in case of a type II odontoid fracture was determined with and without external stabilization using a cervical collar.

## Materials and methods

### Study design

The present biomechanical study was performed on seven unfixed geriatric human cadavers. The study was approved by the local ethics committee (Mainz, Germany; ID: 837.156.16).

Only unfixed geriatric human cadavers that met the following criteria were included in the study: (1) age of 70 years or older, (2) signed informed consent for body donation for scientific investigation, and (3) no existing cervical spine injury or disease. The complete medical records of all body donors were studied and evaluated for suitability.

The unfixed geriatric human cadavers were provided by a body donor program of the Institute for Anatomy and Cell Biology (Heidelberg University, Germany). The body donors were informed, in detail, about the donation and had to give their written consent to body donation during their lifetime. Shortly after the patients’ death, their bodies were frozen. For the present biomechanical study, the unfixed geriatric human cadavers were thawed to room temperature within 24 hours. Experiments were performed on fully preserved unfixed geriatric human cadavers. According to the literature, this process guarantees elasticity of the tissue, joints, and bones that is comparable to those of living humans [40–42].

Inclusion criteria were verified from all donations by reviewing the medical records. Donors with cervical spine disorders, cervical spine surgery in the history and/or cervical spine trauma were excluded from the study.

### Experimental setup

**Fig 1:**
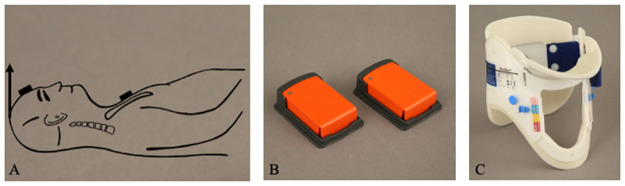
Schematic drawing of the geriatric human cadaver in a supine position (**A**) with the inertial measurement units (IMU) (**B**) fixed to the forehead and sternum. The direction of movement ventrally in the transverse plane is shown (**A** black arrow). External stabilization of the cervical spine was performed using the Ambu Perfit ACE (**C**).

Seven unfixed geriatric human cadavers were included in the present study (four female, three male). The mean age was 82.3 ± 9.4 years (range: 71–94 years). Age, sex, and cause of death are summarized in Table 1. No evidence of previous injuries or diseases of the cervical spine was detected by CT scan. All seven human cadavers had a similar external cervical spine configuration, so the same cervical collar (Ambu^®^ Perfit ACE, Ballerup, Denmark, Fig 1C) could always be applied with the same setting by experienced EMS personnel.

**Table 1:** Listing of individual patients with age, gender, and cause of death.

Patient	Age	Gender	Cause of Death
I	71	♂	Chronic obstructive pulmonary disease
II	75	♂	Acute respiratory distress syndrome
III	94	♂	Sepsis
IV	89	♀	Non-ST-elevation myocardial infarction
V	90	♀	ST-elevation myocardial infarction
VI	85	♀	Non-ST-elevation myocardial infarction
VII	72	♀	Stroke with cerebral hemorrhage

The unfixed geriatric human cadavers were placed in a supine position (Fig 1A). In order to get the cadavers’ bodies fixed during the passive manipulation on the head (movement of the cervical spine), the cadavers were placed on a spine board (Laerdal BaXstrap, Stavanger, Norway) and fixed with a harness fixation system (MIH-Medical Spiderstrap, Georgsmarienhütte, Germany). Thus, relevant artefacts during the measurement could be reduced to an absolute minimum. Three motion measurements were made on the cervical spine of each unfixed human cadaver, as explained below.

To measure the physiological range of motion, the head was then passively moved by flexion and lateral bending to the right in the transverse plane with a tractive force of 100 N. This force is in the same range as the force applied to the cervical spine during intubation [40]. Tractive force was measured with an electronic spring force meter (LENI, Fa. Korona, Sundern, Germany). For the motion measurement the electronic spring force meter was always fixed to the bregma of the unfixed geriatric human cadaver. Ventral movement (Fig 1A black arrow) and lateral movement was standardized at 100 N in the transverse plane.

Afterwards, the surgical procedure was performed to obtain a geriatric type II odontoid fracture model (see description below for details). Again, the head was moved passively as described above (flexion and lateral bending to the right with a tractive force of 100 N) to determine the pathological range of motion in the case of an odontoid fracture.

Finally, the external stabilization of the cervical spine was performed with a cervical collar by experienced EMS personnel. The head was then moved for a third time, and the range of motion in the case of external stabilization of an odontoid fracture was determined.

### Motion measurement

The directions and extent of flexion and lateral bending of the cervical spine were recorded with wireless motion sensors (inertial measurement unit (IMU), Xsens Technologies^®^, Enschede, The Netherlands, Fig 1B). The 3D motion measurement at the cervical spine is registered by two IMUs. The IMUs record all directions of motion (extension, flexion, rotation, and transverse flexion, Fig 3A) during passive movement of the cervical spine (flexion and lateral bending to the right, Fig 1A). Motion measurement with these motion sensors has been tested in various studies [36, 43–45] and guarantees exact measurement results [46]. One IMU were attached to the forehead and the other IMU to the sternum of the unfixed geriatric human cadaver (Fig 1A). The 3D data were synchronized every 20 ms by the recording tool (Xsens Technologies^®^). The data from the recording tool were visualized using Excel software (Microsoft Excel, Version 16.41, Microsoft Corporation, Washington, DC, USA).

### Development of a geriatric type II odontoid fracture model

**Fig 2:**
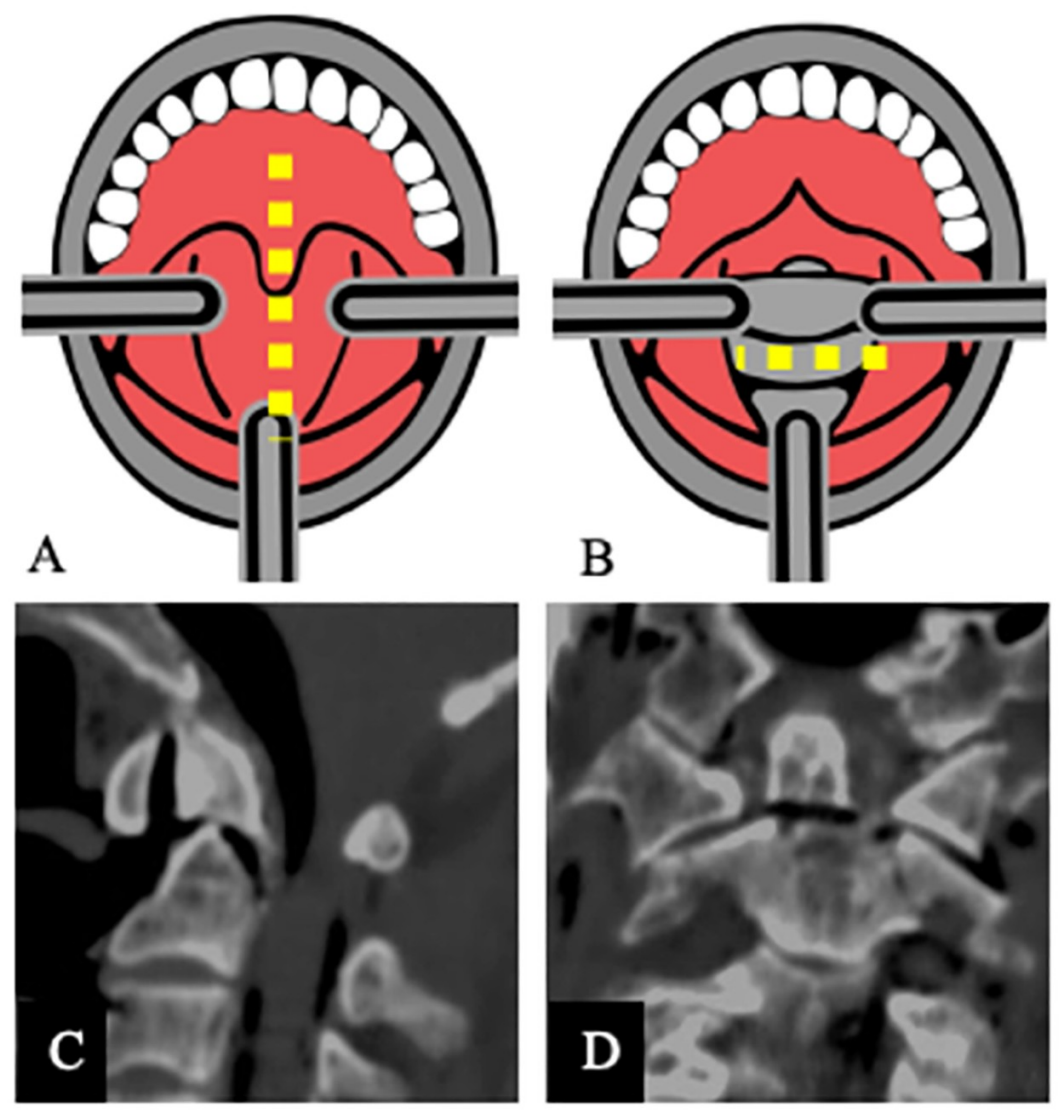
Surgical approach of the geriatric cadaveric trauma model for type II odontoid fracture. The uvula and soft palate were incised (**A**, yellow dotted line). After preparation of the posterior pharyngeal wall, the ventral arch of C1 and the corpus of C2 became visible (**B**). The osteotomy was performed below the ventral arch of C1 ((**B**), yellow dotted line). Sagittal (**C**) and coronal (**D**) computed tomography (CT) image of the geriatric trauma model demonstrating the odontoid fracture is type II.

The surgical procedure was performed on the cadaver in a supine position using a transoral surgical approach to the ventral vertebral bodies C1 and C2 (Fig 2A and 2B). The head was positioned in extension so that the odontoid descended into the surgical field. A retractor was inserted for a good view of the posterior pharyngeal wall (buccopharyngeal fascia and alar fascia). The soft palate was then held away cranially. If the soft palate restricted the approach, it was split in the midline (Fig 2B). The posterior pharyngeal wall was palpated with a needle, and the atlas arch was detected as a prominent structure. The midline was marked using the anterior tubercle of the atlas arch as a landmark. The posterior pharyngeal wall was prepared in the midline. In some non-fixed human preparations, the soft palate and the uvula had to be split (Fig 2B). The muscles were then pushed aside from the clivus, the anterior arch of C1, and the surface of C2. This surgical approach protects all the muscles and ligaments that stabilize the C1-C2 segment. A horizontal fracture was placed at the base of the odontoid, below the anterior arch of C1, using an osteotome. The odontoid fracture was controlled primarily with a preparation hook, which was used to identify instability of the odontoid. Furthermore, computed tomography (CT) scan (Siemens Healthcare GmbH, Erlangen, Germany; Fig 2C and 2D) of the cervical spine of each unfixed geriatric human cadaver was performed to check the geriatric type II odontoid. The CT scan was evaluated using the imaging program Horos (HorosTM, version 3.3.6, FOSS, LGPL license at horosproject.org, sponsored by Nimble Co LLC d/b/a Purview, Annapolis, MD, USA). In addition, the CT scan of the cervical spine was used to check for patients with previous injuries or diseases of the cervical spine.

### Statistical data analysis

In spite of an intense literature search, no studies could be found that address the extent nor the type nor the direction of motion that may further harm a patient with a type II dens fracture. Therefore, it is not possible to define a statement about the significant difference between physiologic, traumatized, and immobilized cervical spine motion. Therefore, sample size calculation could not be performed.

The normal distribution of the three groups (physiological motion vs. pathological motion with and without external stabilization) was evaluated using the Shapiro–Wilk test. Parametric, continuous data were analyzed using ANOVA, and nonparametric data using the Kruskal-Wallis test. Multiple comparisons were performed using Tukey’s multiple comparison test. A *p*-value of < 0.05 was considered as statistically significant. For normally distributed continuous values the descriptive data were given as mean and standard deviation. For better representation, the ranges were also specified. The data were analyzed using the analysis software GraphPad Prism (Prism 8, Version 8.2.1, GraphPad software, San Diego, CA, USA).

## Results

### Development of a geriatric type II odontoid fracture model

The new fracture model for geriatric type II odontoid fractures could be established in each of the seven unfixed geriatric human cadavers. Instability of the odontoid was demonstrated in each cadaver by pulling it with a preparation hook. Furthermore, the CT scans were used to confirmed odontoid fractures type II in all seven unfixed geriatric human cadavers (Fig 2C and 2D). According to the subclassification of type II dens fractures by Grauer et al. [4], type IIA fractures were present in 2 cadavers and type IIB fractures in 5 cadavers (Fig 2C).

### Cervical spine motion measurements

**Fig 3:**
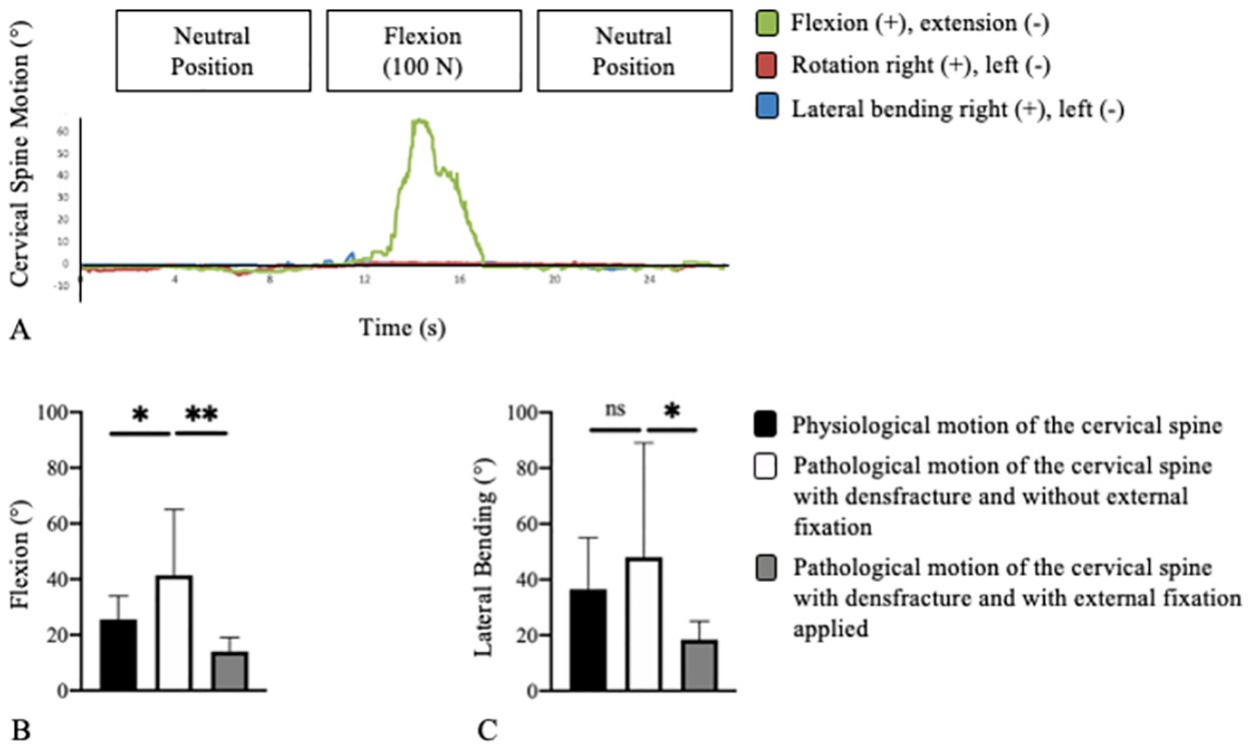
Cervical spine motion of the geriatric cadaveric trauma model without external stabilization during flexion with a traction force of 100 N (**A**). Range of motion of the cervical spine indicated in degrees (°) for flexion (**B**) and lateral bending (**C**).

The mean physiological flexion of the cervical spine of the seven unfixed geriatric human cadavers was 25.6° ± 5.4° (range: 18–34°) and the mean lateral bending to the right was 36.4° ± 8.8° (range: 29–55°). In the cadaveric trauma model of a type II odontoid fracture, flexions of up to 65° were measured (Fig 3A). The mean pathological flexion of the cervical spine in the case of a type II odontoid fracture was increased to 1.6–fold compared to the mean physiological flexion. Thus, the mean flexion was significantly (*p* = 0.027) increased from 25.6° ± 5.4° (range: 18–34°) to 41.4° ± 10.8° (range: 34–65°) in the case of a type II odontoid fracture (Fig 3B). Mean lateral bending was not significantly (*p* = 0.0952) increased in the fracture model (Fig 3C).

In the case of external stabilization of the cervical spine with a type II odontoid fracture, the mean pathological flexion was significantly reduced (*p* = 0.0017) from 41.4° ± 10.8° (range: 34–65°) to 13.9° ± 3.8° (range: 8–19°). Thus, external stabilization of the cervical spine with a type II odontoid fracture reduced motion below physiological range. Furthermore, the mean pathological lateral bending was significantly reduced (*p* = 0.0137) from 48° ± 19.5° (range: 36–89°) to 18.4° ± 3.3° (range: 15–25°).

## Discussion

The aim of the study was to analyze whether the application of a cervical collar can reduce cervical spine motion in geriatric patients with a type II odontoid fracture. For this purpose, In the present study, a standardized cadaveric trauma model for type II odontoid fractures and successfully transferred it to a geriatric patient population was establish. Type II odontoid fractures could be surgically created in seven unfixed geriatric human cadavers as immediately confirmed by an instability test and further confirmed through a CT scan [47]. The advantage of this cadaver model is the easy surgical approach for its implementation and the possibility of simple and quick testing of upper cervical spine instability. According to the current literature, there is still a lack of clarity regarding the best surgical approach for the upper cervical spine [48]. In future, the newly developed model for geriatric type II odontoid fractures will be available to research groups dealing with instability in the upper cervical spine.

According to the literature, the physiological motion of the cervical spine, measured in the present study as flexion and lateral bending, correspond to the typical physiological motion of the cervical spine [49–51]. Cho et al. [52] examined the physiological motion of 16 patients with a mean age of 23.8 years. The authors of that study described a mean flexion of 49° and a mean right-sided tilt of 40°. The range of motion in these results are in a comparable range as in the present study (mean flexion: 26°; mean lateral bending: 36°). Reduced physiological motion of the cervical spine in the study may be age-related [53] and due to the applied force of maximum 100 N. A recent cadaveric study demonstrated that sufficient immobilization of the cervical spine can be achieved with cervical collar even with a geriatric facial structure [54]. The newly developed geriatric type II odontoid fracture model can thus provide important knowledge about the physiological and pathological motion of the cervical spine.

In the newly developed geriatric type II odontoid fracture model, the pathological motion of the cervical spine significantly increased to 1.6–fold compared to the measured physiological motion. Therefore, in the present study of type II geriatric odontoid fractures, it can be assumed that there was instability between the cervical C1 and C2 vertebrae. Neither the musculature nor the ligamentous structures of the upper cervical spine were destabilized in the present study. However, no muscle tone is present when examining human cadavers, only basic ligamentous tension. Some authors have already observed the possibility of this instability in type II odontoid fractures and have established more exact subclassifications [4, 55].

In the case of an unstable injury to the upper cervical spine, secondary damage can occur during necessary manipulations of the patient’s head during emergency care [31, 35]. Emergency airway management can contribute to cervical intervertebral motion and may aggravate cervical spinal cord injury in the presence of an unstable cervical spine [40, 56]. Therefore, immediate external stabilization of the cervical spine should be considered. Häske et al. were able to demonstrate that if a person is rescued from a car, an attached external stabilization of the cervical spine should always be performed since additional motion of the cervical spine can be reduced [43]. The same conclusion was reached in another study, by Nolte et al., concerning patient transport in an ambulance [44].

According to the data from the current study, external stabilization in geriatric patients should still be recommended if an odontoid fracture is suspected. This recommendation is supported by several other studies in which the authors appeal for external stabilization of the cervical spine as spinal trauma cannot be excluded until the final diagnosis in the trauma center [16–20]. Based on the results of the current study, prehospital treatment without external stabilization may be discouraged [32, 57] since cervical spine motion increases significantly due to instability. Cervical spine instability can lead to serious complications, as described above [31, 35].

However, the application of external stabilization also has some negative effects. Disadvantages such as pain, discomfort [23, 24], or pressure ulcers [23, 25, 26] have been described. In addition, restrictions in respiratory function [27], difficult airway management [28], increased intracranial pressure [29, 30], and even compression of the dural sac [31] have been described, which can lead to severe complications, particularly in geriatric trauma patients.

Due to these considerable disadvantages, external stabilization of the cervical spine using a cervical collar is not a long-term solution. Further studies must show if other immobilization techniques can supply sufficient immobilization while reducing negative side effects. Recent studies have shown that adequate immobilization of the cervical spine may also be achievable without using a cervical collar [36, 58]. Furthermore, studies should compare the effects of an incorrectly applied cervical collar on the motion of an injured upper cervical spine. Ben-Galin et al. found severe distraction between C1 and C2 during the placement of a cervical brace in their trauma model [59]. However, comparison with this study is difficult because, unlike the present study, both the bony and ligamentous structures were transected. In particular, user variability and quality of cervical collar application and contact strength should be considered. Recent studies have been able to show that the application of a cervical collar causes immense movement of the cervical spine [31, 35].

## Limitations

In the present study, a new geriatric fracture model could be developed. However, this model has not yet been validated. Since cadaver models lack of any muscle tone, the results may only be comparable to patients with a deep loss of consciousness. The transferability of the current model to patients with some muscular tone left is unclear. Thus, further studies must show to what extent the new model can be transferred to patients.

In the present study, palpation hooks and CT scan were used to check osteotomy and instability. Additional fluoroscopy that may give additional information on the amount of instability was not performed. Only the motion of the entire cervical spine can be evaluated by the experimental setup described above. An isolated examination of the C1/C2 segment would be desirable.

## Conclusions

A geriatric trauma model for type II odontoid fractures was established. It was found that type II odontoid fracture significantly increases the motion of the geriatric cervical spine. External stabilization using a cervical collar can significantly restrict this motion in geriatric cadavers with type II odontoid fractures.

## Supporting information

S1 File (PDF)
