# Supplementary material for: Evaluation of external stabilization of type II odontoid fractures in geriatric patients—An experimental study on a newly developed cadaveric trauma model
Source: PLoS One. 2021 Nov 29;16(11):e0260414. doi: 10.1371/journal.pone.0260414 (PMC8629171; doi:10.1371/journal.pone.0260414)
Supplement: S1 File — (PDF) [file pone.0260414.s001.pdf]

Cadaver/Motion	P	PH WO EF	PA W EF	P	PH W EF	PA W EF
1	29	36	19	25	34	17
2	35	56	25	31	43	11
3	55	89	19	34	37	16
4	35	45	15	25	65	14
5	29	36	17	23	36	12
6	36	38	16	23	39	19
7	36	36	18	18	36	8
